# RNA Chemical Modifications in Mammalian Skeletal Muscle Development, Homeostasis, and Disease: Regulatory Mechanisms and Chemical Biology Perspectives

**DOI:** 10.3390/molecules31162797

**Published:** 2026-08-11

**Authors:** Dujun Chen, Mailin Gan, Yuhang Lei, Xinyi Wang, Jincheng Zan, Qing Ye, Xiaofeng Zhou, Lei Chen, Yan Wang, Ye Zhao, Li Zhu, Linyuan Shen

**Affiliations:** 1 Farm Animal Germplasm Resources and Biotech Breeding Key Laboratory of Sichuan Province, Sichuan Agricultural University, Chengdu 611130, China; 2024202034@stu.sicau.edu.cn (D.C.); ganmailin@sicau.edu.cn (M.G.); leiyuhng@gmail.com (Y.L.); 2024202054@stu.sicau.edu.cn (X.W.); zanjincheng@stu.sicau.edu.cn (J.Z.); yeqing@stu.sicau.edu.cn (Q.Y.); zxf93715@163.com (X.Z.); chenlei815918@sicau.edu.cn (L.C.); 14916@sicau.edu.cn (Y.W.); zhye@sicau.edu.cn (Y.Z.); 2 State Key Laboratory of Swine and Poultry Breeding Industry, Sichuan Agricultural University, Chengdu 611130, China; 3 Key Laboratory of Livestock and Poultry Multi-Omics, Ministry of Agriculture and Rural Affairs, College of Animal and Technology, Sichuan Agricultural University, Chengdu 611130, China

**Keywords:** RNA modification, skeletal muscle, dynamic regulation, m6A

## Abstract

Skeletal muscle is a core component of the motor system, responsible for movement, posture maintenance, and energy metabolism. It plays a vital role in physiological stress, injury repair, and metabolic homeostasis. In recent years, with the advancement of epigenetic research, RNA modification has become a hot topic in skeletal muscle biology. This article systematically reviews the progress in the study of RNA modification in regulating the fate of skeletal muscle, focusing on the dynamic regulatory mechanisms and pathophysiological significance of N6-methyladenosine (m^6^A) modification in muscle differentiation, regeneration, and atrophy. It also briefly introduces the existing evidence for 5-methylcytidine (m^5^C), adenosine-to-inosine (A-to-I) editing, and pseudouridine (Ψ) in skeletal muscle. A comprehensive understanding of these mechanisms is crucial for elucidating the physiological regulation of skeletal muscle and the pathogenesis of related diseases and also provides a new direction for the development of intervention strategies.

## 1. Introduction

Skeletal muscle is one of the largest organs in the body, accounting for about 40% to 50% of an adult’s weight. Its main functions include movement, posture maintenance, and metabolic regulation [1]. The growth and development of skeletal muscle is a continuous, coordinated biological process involving the proliferation and differentiation of myoblasts, the formation of myotubes, and their regeneration and fusion [2,3,4]. In a resting state, satellite cells are stimulated and then activated, enter the cell cycle, subsequently proliferating, differentiating, and fusing to form new muscle fibers or repair damaged ones, maintaining the stability of the muscle structure and function [5,6]. Muscular atrophy is commonly seen in muscular dystrophy, aging, and neuromuscular diseases. At this time, the regeneration ability is impaired, the number of muscle fibers decreases, and the muscle mass and function decline [7,8,9].

Post-transcriptional RNA modification is an important component of epitranscriptomics and one of the key links in regulating the fate of skeletal muscle cells [10,11]. These modifications regulate the stability, splicing, transport, and translation efficiency of RNA, and then participate in the processes of muscle proliferation, differentiation, regeneration, and atrophy [10]. Currently, several RNA modifications, such as N^6^-methyladenosine (m^6^A), 5-methylcytosine (m^5^C), N^1^-methyladenosine (m1A), N^7^-methylguanosine (m7G), and N^4^-acetylcytidine (ac4C), have been extensively studied and have clear functions [12,13]. Despite the increasing number of related studies, the functional boundaries, synergistic relationships, and pathological significance of different RNA modifications in skeletal muscles are still poorly understood.

Therefore, this review paper focuses on the enzymatic basis, detection strategies, key targets, and signal pathways of RNA modification, highlighting its regulatory role in skeletal muscle physiology and diseases, with a particular focus on the dynamic mechanisms and functional significance of m^6^A.

## 2. Overview of RNA Modifications

RNA modifications occur on the bases or the sugar-phosphate backbone of RNA. With the help of specific enzymes, chemical groups are attached to create unconventional nucleotide structures. These structures significantly alter the physicochemical properties and downstream functions of RNA. RNA modifications are widely found in mRNA, tRNA, rRNA, and non-coding RNAs (such as lncRNA and miRNA), forming an important part of the post-transcriptional regulatory network [14,15]. More than 170 types of modifications are known. m^6^A, m^5^C, and A-to-I editing have received extensive attention due to their well-defined functions [11,16].

For example, in m^6^A, the methyltransferase like 3 (METTL3)- methyltransferase like 14 (METTL14)-WT1 associated protein (WTAP) complex is responsible for methylation, demethylation enzymes such as FTO (Fat mass and obesity-associated protein) and alkB homolog 5 (ALKBH5) are responsible for removing the modification, and recognition proteins such as YTH N6-methyladenosine RNA binding protein 1-3 (YTHDF1-3) and YTH domain containing 1 (YTHDC1) recognize the m^6^A site and further affect mRNA splicing, stability, nuclear export, and translational efficiency [17,18,19,20,21]. m^5^C is mainly written by NOP2/Sun RNA methyltransferase 2 (NSUN2) and recognized by recognition proteins such as Aly/REF export factor (ALYREF), and is involved in RNA export, localization, and myogenic-related processes [22]. Most studies on modifications such as m1A, m7G, and ac4C have focused on tRNA and rRNA. They are mainly involved in translation accuracy, protein synthesis, and RNA stability, but their specific functions in skeletal muscle are unclear [10,23].

### 2.1. Types of RNA Modifications

m^6^A modification is one of the most abundant modifications on RNAs, referring to the methylation of the adenine (A) base at the N6 position, a methyl group (–CH_3_) is attached to the 6th nitrogen atom of the adenine. It occurs widely in mRNA, long non-coding RNAs (lncRNAs), and circular RNAs (circRNAs). On mRNA, it is mainly concentrated in the 5′ UTR, the coding region, and the 3′ UTR, with denser concentrations near the stop codon [24,25,26]. In skeletal muscle, the level of m^6^A modification varies significantly across developmental stages and between different muscle fiber types, suggesting that m^6^A plays an important role in muscle development and functional maintenance [10,24]. Data have shown that m^6^A dynamically regulates the fate of muscle satellite cells through proteins such as METTL3/14, FTO/ALKBH5, and YTHDF [27,28]. It also regulates the expression of major myogenic transcription factors such as myogenic differentiation 1 (*MYOD1*) and myogenin (*MYOG*), influencing satellite cell activation, proliferation, and differentiation [25,29]. The HNRNPA2B1 protein mediates the fate transition of myogenic cells by regulating specific intrinsic and downstream splicing regulators [30].

m^5^C modification, which mainly occurs in tRNA and rRNA and has also been found in mRNA, refers to the methylation of the fifth carbon atom of the cytosine (C) base in RNA molecules. It mainly affects RNA stability and translation efficiency [13].

A-to-I editing is a classic type of RNA editing catalyzed by ADAR family proteins. It refers to the deamination of adenosine (A) in RNA molecules, converting the 6th amino group (-NH_2_) to a carbonyl group (=O), thereby producing inosine (I). This process alters base pairing, RNA secondary structure, and translation potential [10,13]. This process is involved in RNA splicing and the regulation of non-coding RNA function and may also serve in adaptive regulation under oxidative stress and metabolic changes [11]. During skeletal muscle repair, changes in Adenosine Deaminase Acting on RNA 1 (ADAR1) levels are closely related to stem cell proliferation and differentiation.

In addition, RNA modifications include m1A, m^7^G, and pseudouridine (Ψ). m^1^A is found in various RNA species including tRNAs, rRNAs, and mRNAs, influencing post-transcriptional modification rates and translational efficiency [14,31]. m^7^G modification is located in the cap structure of RNA and also occurs as an internal modification in mRNA, it is a key regulator of mRNA translation initiation [32]. Pseudouridine (Ψ) is a modified nucleotide formed by base isomerization of uridine (U) in RNA molecules, in which the bond between the uridine base and the ribose changes from the conventional C-N bond to a C-C bond. It plays an important role in RNA maturation and splicing by altering RNA structure and stability [13]. These modifications together form a complex epigenetic regulatory network, providing the basis for fine-tuning the temporal and spatial expression of RNA functions.

### 2.2. The “Write-Erase-Read” Dynamic Regulatory System of RNA Modifications

There are three types of proteins that dynamically regulate RNA modifications: methyltransferases (writers), demethylases (erasers), and modification-recognition proteins (readers) [29,33]. Methyltransferases add chemical groups to RNA, demethylases remove chemical groups from RNA, and recognition proteins recognize and bind to RNA modifications.

Some common RNA modifications and their corresponding writers, readers and erasers are summarized in Table 1. Note that there are many RNA modifications not listed in the table.

### 2.3. RNA Modification Detection Technologies

Skeletal muscle is composed of multiple cell types, including satellite cells (MuSCs), myoblast precursor cells, immune cells, fibro-adipocyte precursor cells (FAPs), and muscle fibers. It also has numerous blood vessels and Schwann cells surrounding axons. They work together in space and time to maintain homeostasis and repair injury [62,63,64]. The complexity of this cellular assembly makes post-transcriptional regulation, particularly RNA modifications, highly temporally and spatially specific during muscle development, regeneration, and atrophy. However, it is difficult for traditional bulk sequencing to capture rare cell subsets or modifications in the local microenvironment [34,65].

RNA modification detection technologies have evolved. Early Methylated RNA Immunoprecipitation Sequencing (MeRIP-seq) and m^6^A-seq methods relied on antibodies to capture m^6^A-rich RNA fragments, then immunoprecipitated, high-throughput sequenced them, and found the methylation regions through peak recognition, a process that provided the basis for full transcriptome analysis [66]. However, these methods had limited resolution in their localization and significant false-positive and false-negative issues, and required large starting samples [34,65,67]. Later, single-base resolution methods emerged, such as Deamination adjacent to RNA modification targets (DART-seq) and Glyoxal and nitrite-mediated deamination of unmethylated adenosine (GLORI). DART-seq, based on the APOBEC1–YTH fusion protein, is an enzyme-assisted strategy, while GLORI uses a purely chemical strategy, this single-base resolution method greatly improving site accuracy and reducing sample requirements [68,69,70,71]. DART-seq uses a fusion protein consisting of the APOBEC1 cytidine deaminase and the m^6^A-binding YTH domain (APOBEC1-YTH) fusion protein to convert cytosine (C) next to m^6^A to uracil (U), which is then read by standard RNA–seq, allowing researchers to accurately locate the position of each m^6^A, down to individual bases [69,72]. This method can be performed in living cells without destroying RNA integrity and identifies m^6^A subtypes at the single-cell level [73,74,75]. GLORI uses a purely chemical method. It first uses glyoxal to protect guanine (G) and then selectively deaminates unmethylated adenine (A) to inosine (read as G during sequencing). m^6^A is not affected and is still read as A. This allows absolute quantification at single-base resolution on a standard Next-Generation Sequencing (NGS) platform [68]. These methods have pushed the localization of m^6^A to a higher level of precision.

Furthermore, single-cell technologies are gradually advancing. Single-cell transcriptome sequencing (scRNA-seq) is a common method for analyzing cell heterogeneity, with most studies conducted on the 10× Genomics Chromium platform. This platform employs droplet microfluidic technology: individual cells are labeled with barcode-bearing gel microbeads, and reverse transcription, library construction, and sequencing are used to obtain each cell’s gene expression profile [76,77]. However, this method requires high-quality single-cell suspensions made from fresh tissue. To address this limitation, single-nucleus transcriptome sequencing (snRNA-seq) was developed, which does not require dissociating tissue. Instead, nuclei are released from cells using a detergent, and these nuclei are then subjected to RNA sequencing snRNA-seq also works with frozen and FFPE samples [78]. Additionally, when studying complex tissues like the brain, snRNA-seq has become more common than scRNA-seq [77]. Single-cell and single-nucleus transcriptomics studies have revealed that resting satellite cells are molecularly heterogeneous. Different subsets have different activation thresholds and fate tendencies. This hierarchical structure provides a precise target for post-transcriptional modifications [63,79,80]. The activity of RNA modification enzymes such as METTL3 and protein readers such as YTHDC1 is closely related to the transition from resting to active states of MuSCs [18,81]. In mature muscle fibers, the expression levels of RNA modification enzymes vary among different subtypes, suggesting that the modification layer may regulate fiber-specific metabolic and translational processes. In senescence or pathological states, abnormal modifications may preferentially destroy the maintenance mechanisms of specific fiber subtypes, impairing muscle function [64,82]. These advances have deepened our understanding of skeletal muscle biology.

## 3. m^6^A Regulates Skeletal Muscle Growth and Development

The growth and development of skeletal muscle is an orderly and dynamic biological process, involving stages of myocyte proliferation, differentiation, and fusion. During embryonic development, myoblasts activate myogenic transcription factors such as MYOD1, myogenic factor 5 (*MYF5*), MYOG, and muscle regulatory factor 4 (*MRF4*) to initiate the myogenic program [83,84]. As an epigenetic regulatory mechanism, RNA modification influences skeletal muscle growth and development by regulating RNA stability, translation efficiency, splicing, and localization. Studies have focused on m^6^A and m^5^C modifications, which are involved in the differentiation of myoblasts, the formation of muscle fibers, and muscle regeneration by regulating the expression of multiple genes [22,85,86,87].

### 3.1. Effects of m^6^A on the Proliferation of Myogenic Progenitor Cells During Embryogenesis

Embryonic skeletal muscle development begins with myogenic progenitor cells (MPCs), which proliferate, migrate, and differentiate in an orderly manner under the guidance of transcription factors such as paired box 3 (*PAX3*) and paired box 7 (*PAX7*) [88,89]. This process is characterized by rapid cell proliferation and differentiation, which requires high protein synthesis. The dynamic changes in m^6^A modification patterns are highly synchronized with the expression of myogenic genes such as *MYOD1* and *MYOG*, indicating that m^6^A modification plays a crucial role in the muscle development timeline [90]. By regulating the mRNA stability and translation efficiency of these transcription factors, m^6^A directly affects the proliferation and differentiation abilities of MPCs [29,33].

METTL3 enhances translation efficiency by adding modification groups to the *MYOD1* mRNA, YTHDF1 recognizes the m^6^A modification [17,91]. Deletion of METTL3 results in decreased *MYOD1* protein levels and developmental disorders in muscle development [91,92]. In addition to directly regulating myogenic transcription factors, METTL3 maintains the proliferative state of progenitor cells and prevents premature differentiation by modifying key factors of the Notch pathway, such as Notch receptor 2 (*Notch2*) and recombination signal binding protein for immunoglobulin kappa J region (*RBPJ*) [93]. Heterogeneous nuclear ribonucleoprotein A2/B1 (HNRNPA2B1) is an m^6^A recognition protein that recognizes m^6^A-modified RNAs, regulating their splicing, output, and degradation [94,95]. It indirectly promotes progenitor cell proliferation by regulating the splicing of muscle-related pri-miRNAs [96]. Another recognition protein, IGF2BP3, also plays an important role. Liu et al. found that IGF2BP3 stabilizes calcium/calmodulin dependent protein kinase II beta (*CAMK2B*) mRNA, activates myocyte enhancer factor 2C (MEF2C) signaling pathway, and drives muscle differentiation [97].

In goat myoblasts, FTO enhances the stability and expression of target mRNAs such as growth arrest and DNA damage inducible beta (*GADD45B*) and cyclin D1 (*CCND1*) by removing m^6^A modifications [98,99]. FTO also demethylates transforming growth factor beta 1 (*TGFβ1*), activating the SMAD family member 2 (SMAD2) signaling pathway and promoting proliferation [100]. This ensures adequate proliferation and expansion of myogenic progenitor cells after injury or during development, providing a sufficient cellular base for subsequent normal skeletal muscle formation and regeneration. Additionally, m^6^A can synergistically regulate embryonic muscle development with the miRNA network [101].

### 3.2. The Impact of m^6^A on Myoblast Differentiation and Myotube Formation

Myoblast differentiation is a crucial stage in the development of skeletal muscle. During this process, myoblasts exit the cell cycle, initiating differentiation, fusing into multinucleated muscle myotubes, and then forming mature muscle fibers. During this differentiation, RNA modifications finely regulate this process through multiple mechanisms, ensuring the normal development and optimal function of skeletal muscle.

METTL3 promotes mRNA translation of myogenic regulators such as myosin heavy chain gene (*MYH*), *MYOD1*, and *MYOG* via YTHDF1, which in turn drives myoblast differentiation [17]. METTL3 also directly regulates key myogenic factors, *MYOD1* and *MEF2C*, and enhances translation efficiency through YTHDF1 to ensure the initiation of the differentiation program [91,102,103]. During the growth and development of skeletal muscle, m6A modifications also un-dergo dynamic changes [104]. In adult muscle fibers, METTL3 inhibits the translation of activin A receptor type 2A (*ACVR2A*) via m^6^A modifications, thereby removing anti-hypertrophic signals and maintaining muscle fiber size and supporting overload growth [92]. METTL3/METTL14 also regulate the expression of muscle-specific miRNAs and lncRNAs, which together control the differentiation program [105,106,107,108].

Different readers decode the same chemical modification into different functional outputs. YTHDF1 and METTL3 synergistically enhance the translation efficiency of myogenic factors such as *MYOD1* [17]. YTHDF2 selectively degrades proliferator-related transcripts (such as serine/threonine kinase 11 (*STK11*)), removing the inhibition of the mechanistic target of rapamycin (mTOR) pathway and promoting the proliferation-differentiation state transition [109]. Nuclear-positioned YTHDC1 recognizes the m^6^A tags on *MYOD1* and *MYOG*, facilitating their nuclear transfer and accelerating myoblast differentiation [110]. In the insulin like growth factor 2 mRNA binding protein (IGF2BP) family, IGF2BP1 inhibits skeletal muscle differentiation by regulating the fibroblast growth factor receptor 1 (FGFR1)-extracellular signal-regulated kinase (ERK) axis [111]. IGF2BP3 stabilizes the transcription of *CAMK2B* to activate the downstream *MEF2C* cascade [97]. In addition, the pre-miR-503-5p is specifically recognized by readers and subsequently activates the mTOR pathway, directly linking RNA modification to growth signals, expanding the functions of m^6^A in non-coding RNA species [107]. This ‘same modification, multiple interpretation’ mechanism enables the m^6^A system to regulate differentiation at different stages.

FTO and ALKBH1/5 are dualistic in myoblast differentiation, exhibiting situational dependence and functional bidirectionality. Studies have shown that FTO activates p38 mitogen-activated protein kinase (p38 MAPK) signaling by removing m^6^A modifications on *GADD45B*, thereby promoting differentiation [99]. It also enhances the stability of dystroglycan 1 (*DAG1*) mRNA, which promotes proliferation [112]. However, FTO can also promote differentiation by regulating mitochondrial biosynthesis through the mechanistic target of rapamycin–peroxisome proliferator-activated receptor gamma coactivator 1-alpha (mTOR-PGC-1α) axis [20]. In contrast, ALKBH1 inhibits differentiation by influencing DNA methylation and downstream gene networks [113].

### 3.3. The Regulatory Role of m^6^A in Muscle Fiber Type Conversion and Metabolic Characteristics

Mature skeletal muscles are composed of various types of muscle fibers, including slow-twitch (Type I) and fast-twitch (Type II) fibers [4]. Slow-twitch fibers mainly engage in aerobic metabolism, are rich in mitochondria, and are resistant to fatigue. Fast-twitch fibers, on the other hand, are mainly anaerobic, contract quickly, and fatigue easily. The types of muscle fibers and their metabolic characteristics determine the diversity and adaptability of skeletal muscle functions [114,115]. The varying proportions of these muscle fiber types affect athletic performance and are closely linked to multiple metabolic diseases, aging, and pathological conditions such as muscle atrophy [116,117].

#### 3.3.1. m^6^A Regulates Myofiber Type Conversion

The conversion of muscle fiber types depends on a comprehensive reconstruction of the underlying transcriptional network. The m^6^A modification establishes a clear molecular regulatory chain in this process: upstream modifiers (e.g., METTL3), midstream target RNAs, and downstream effector pathways. Upstream modifiers such as writers, readers, and erasers have significantly different expression and activity in fast and slow muscle fibers [115,118]. These modifiers determine the direction of fast-slow muscle conversion by targeting specific transcripts. For example, m^6^A modifications have been shown to regulate the expression of myogenic transcription factors such as *MYOG*, and *MYOG* transcripts are more highly expressed in slow muscles [17,119]. METTL3 can also stabilize growth hormone receptor (GHR) mRNA through m6A modification, promoting its translation and indirectly influencing fiber type conversion [120]. The METTL3 complex can recognize the m^6^A site of Glycerophosphocholine phosphodiesterase 1 (*GPCPD1*) and drive the conversion of porcine muscle fibers [114]. m^6^A modification can also target the slow muscle-specific structural gene myosin heavy chain 7 (*Myh7*), enhancing its mRNA stability and translation efficiency and promoting the conversion of skeletal muscle from fast-twitch to slow-twitch fibers [121,122]. In addition, FTO is also involved in positive regulation by removing the m^6^A modification from nuclear factor of activated T cells 1 (*NFATC1*) mRNA, thus relieving post-transcriptional inhibition and promoting the formation of slow muscle fibers [117]. METTL16 is involved in inhibiting differentiation and promoting the formation of slow muscle fibers [123].

#### 3.3.2. m^6^A Regulates Muscle Fiber Metabolic Characteristics

The metabolic characteristics of muscle fibers, which are determined by the phenotype associated with the type transition, also determine the endurance and contraction characteristics of skeletal muscles. There are differences in the m^6^A modification patterns between the oxidative/slow and glycolytic/fast skeletal muscles of pigs. m^6^A inhibits the lncRNA MSTRG.14200.1, which delays the differentiation of satellite cells and promotes the transition from fast to slow muscle fibers [124]. m^6^A also regulates the alternative splicing of precursor mRNA and is involved in the formation of phenotypic differences between slow and fast muscles [125]. More importantly, RNA modification provides a key sensing interface for skeletal muscle metabolism to respond to environmental and nutritional stresses. The exogenous nutrient factor, docosahexaenoic acid (DHA), can up-regulate the expression of FTO, systematically reducing the overall m^6^A modification level of muscle cells, and upregulate both DNA damage inducible transcript 4 (*DDIT4*) and the core metabolic regulator *PGC-1α* [126]. This enhances mitochondrial biosynthesis and fatty acid oxidation, and also inhibits the abnormal transition from slow-twitch to fast-twitch fibers induced by high-fat diets, maintaining metabolic homeostasis.

In addition to directly regulating protein-coding genes, RNA modification indirectly affects metabolic networks through non-coding RNA pathways. For example, the modification of pre-miR-503-5p by m^6^A can promote the maturation of miR-503-5p, which then specifically activates the mTOR pathway, promoting the shift of skeletal muscle toward oxidative metabolism and slow muscle fiber type specification [107]. This creates a new mechanism of ‘modification-non-coding RNA-metabolic pathways’. Core enzymes like METTL3 and FTO have been shown to have the ability to reshape muscle fiber composition. By down-regulating or over-expressing core enzymes such as METTL3 and FTO, researchers can reshape the fiber composition [115]. These regulated transcripts then converge on downstream effector pathways, activating the mTOR, PI3K/AKT, NFATC1, and PGC-1α-related mitochondrial biogenesis-related axes, completing the transformation from molecular markers to cellular phenotypes [104,109,123].

### 3.4. Regulatory Characteristics of m^6^A on Myogenic Factors in a Developmental Context

After summarizing the previous mechanisms, it is apparent that m^6^A modification in skeletal muscle development acts more like a one-way timer. Its core function is not simply to activate or enhance myogenesis but to guide proliferating progenitor cells towards final differentiation through an irreversible sequence of modifications.

This process is divided into three steps. In the developmental window, the writer labels the transcripts of key myogenic factors with m^6^A [17,92]. The reader stabilizes the transcripts, enhancing translation efficiency and promoting the expression of differentiation-promoting factors beyond the threshold, creating positive feedback [97]. Subsequently, the eraser activity is downregulated, further consolidating the differentiation direction, and the overall transcriptome stabilizes after differentiation is initiated [98].

The same enzyme (such as METTL3) can have different regulatory effects during proliferation and differentiation phases [127]. The reason may not be changes in the enzyme activity itself, but rather differences in the downstream decoding methods at different developmental stages. This ‘timer’ not only works in the post-transcriptional phase, but is also embedded in upstream developmental signal networks such as Notch and TGF-β, jointly determining postnatal muscle mass and fiber type specialization [100,128].

A key feature of this timer is its one-way nature. Once the developmental window closes, it is difficult to alter the entire epigenome by simply interfering with one enzyme. This insight helps to understand the differences in m^6^A modification regulation across different states and provides a basis for further comparisons of the regulatory logic in regenerative and pathological conditions [103]. 

**Figure 1:**
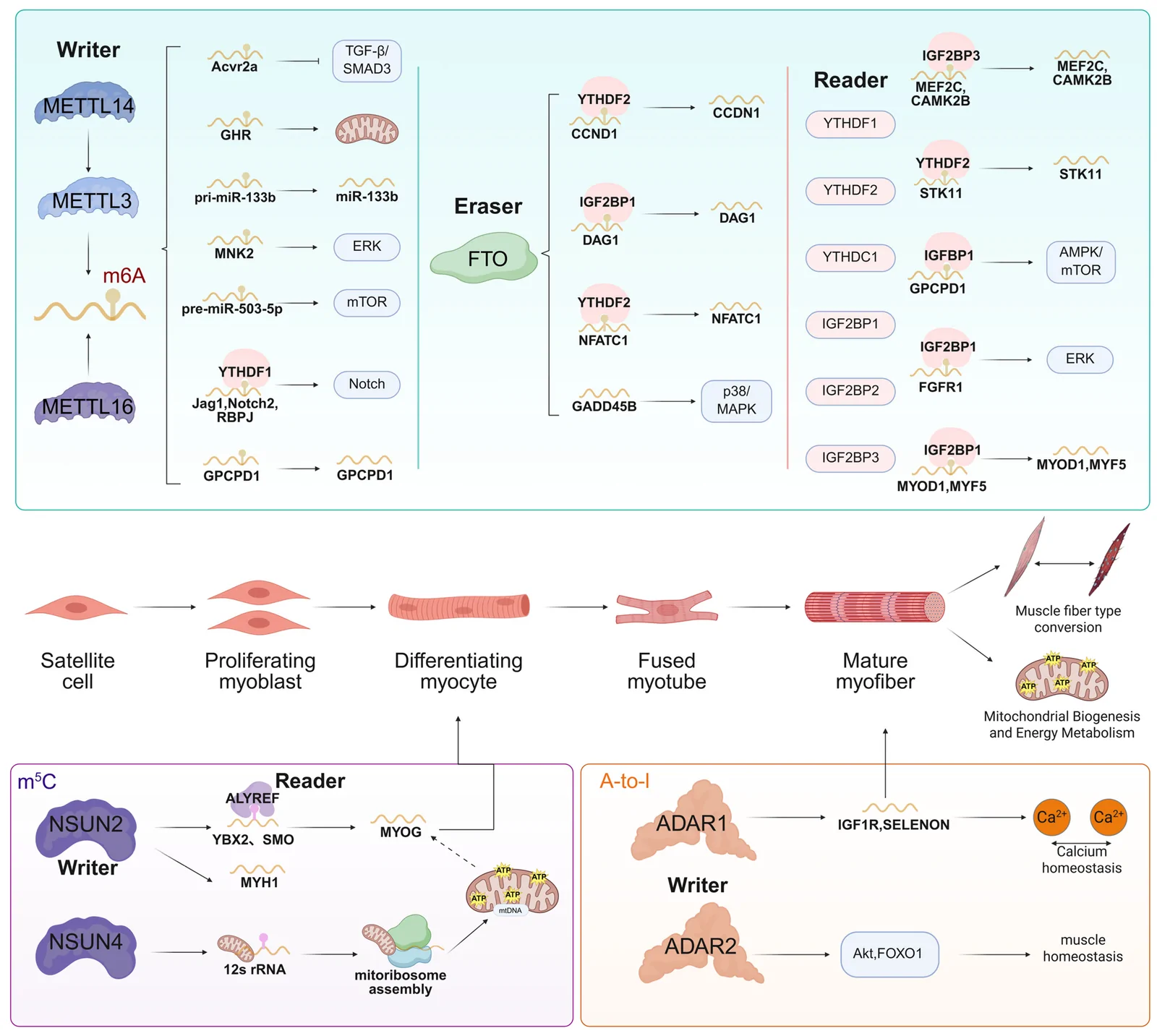
RNA modification pathways in muscle development and metabolism. In m^6^A modification, writer, reader, and eraser jointly affect transcripts such as Acvr2a, pri-miR-133b, growth hormone receptor (GHR), MAPK-interacting kinase 2 (MNK2), and regulate the proliferation, differentiation, and fusion of myoblasts into mature muscle fibers through down-stream MYOD1, MEF2C, and AMPK/mTOR signals. In the m^5^C pathway, NSUN2 targets Y-box binding protein (YBX), smoothened (SMO), MYOG and myosin heavy chain 1 (MYH1), NOP2/Sun RNA methyltransferase 4 (NSUN4) is involved in 12S rRNA assembly, and factors such as ALYREF synergistically promote differentiation. The A-to-I editing is catalyzed by ADAR1/2, and calcium homeostasis is maintained by insulin-like growth factor 1 receptor (IGF1R) and selenoprotein N (SELENON). The light green area in the figure is labeled m^6^A, with color-coded dividers separating the three functional modules: Writer, Eraser, and Reader. The purple dotted box in the lower left corner is labeled m^5^C, and the light orange area in the lower right corner is labeled A-to-I, where ‘→’ stands for ‘positive’ and ‘┤’ for ‘inhibition’.

Figure 1 summarizes these findings, and also depicts the mechanisms by which m^5^C and A-to-I editing regulate skeletal muscle.

## 4. m^6^A Regulates Skeletal Muscle Regeneration

Skeletal muscle regeneration is a complex and finely regulated biological process. It mainly involves the activation, proliferation, differentiation, and reintegration of muscle precursor cells (such as satellite cells) to form new multinucleated muscle fibers [10,62]. During this process, RNA modifications play a significant role at the epigenome level. Studies have shown that RNA modifications such as m^6^A, m^5^C, and A-to-I can influence the fate and function of muscle precursor cells by regulating the stability, splicing, nuclear export, and translation efficiency of key genes [10,11]. The dynamic changes in these RNA modifications ensure the efficient regeneration and repair of skeletal muscles in response to injuries, diseases, or physiological adaptations [10,129].

### 4.1. m^6^A in Muscle Cells During the Regeneration and Reintegration Phases

Regeneration of skeletal muscle after injury relies on activating the satellite cells (MuSCs) from a resting state. The dynamic reprogramming of the m^6^A modification system plays an important role in this process.

After muscle injury, quiescent satellite cells are activated and rapidly proliferate. At this time, the expression of METTL3 is up-regulated, resulting in an increased overall level of m^6^A modification [92,127,130]. METTL3 activates multiple regeneration signaling pathways through differential modifications of various targets: depositing m^6^A on the Notch receptor 1 (*Notch1*) mRNA to enhance stem cell activation and self-renewal driven by Notch signaling [93]; depositing m^6^A on the *MNK2* mRNA to to stabilize the transcript and activate ERK signaling, thereby driving myogenic dif-ferentiation and skeletal muscle regeneration [131]. RNA-binding proteins such as IGF2BP2/3 regulate stress responses and the expression of regeneration-related genes by recognizing these modifications [132,133]. In addition, METTL3 integrates the m^6^A system and non-coding RNA networks by regulating the expression and processing of the circular RNA of phosphatidylinositol-binding clathrin assembly protein (circPICALM)/miR-132/phosphorylase kinase regulatory subunit beta (PHKB)axis and muscle-specific miRNAs [105,134,135]. Furthermore, METTL14 plays a unique role in skeletal muscle regeneration. It targets the regulation of miR-133b and inhibits anti-differentiation genes, promoting differentiation in concert with METTL3 [108].

YTHDC1 recognizes the m^6^A modifications on phosphatidylinositol 4-kinase beta (*PI4KB*) and NIMA related kinase 1 (*Nek1*) mRNA, promotes their splicing and nuclear export, and activates the phosphatidylinositol 4-kinase (PI4K)/protein kinase B (AKT)/mTOR pathway, accelerating the proliferation of stem cells [18,19]. YTHDF2 guides the mRNA of transmembrane 4 L six family member 1 (*TM4SF1*) into the P-body for degradation, preventing abnormal cell proliferation caused by excessive accumulation of untranslated mRNA [136]. IGF2BP3 forms a complex with lin-28 homolog A (Lin28a), recognizes the m^6^A modifications of target genes such as ubiquitin specific peptidase 1 (*Usp1*), and stabilizes the mRNA of stress-responsive genes through the de-ubiquitination pathway, supporting regeneration [132]. It also up-regulates the expression of cell cycle-related genes, directly driving the cell cycle progression [133]. Other regulatory factors, such as FTO, stabilize the mRNA of transcription factors such as *MYOD1* and *MYOG*, by removing m^6^A modifications, promoting the differentiation of muscle cells [137]. When the function of FTO is inhibited, regeneration is significantly delayed, suggesting that eraser activity is necessary for the advancement of the regeneration timing [137].

Non-coding RNAs also play a significant role in muscle regeneration. Downregulation of miR-204 promotes skeletal muscle regeneration [138]. lncRNA MAR1, as a sponge of miR-487b, effectively promotes the differentiation and regeneration of skeletal muscle [139]. The expression and functions of these non-coding RNAs are also regulated by RNA modifications [140].

### 4.2. Aberrant m^6^A Modifications in Regenerative Disorders

The core issue in skeletal muscle regeneration is the impaired activation, proliferation, or differentiation of satellite cells, which ultimately leads to the failure of injury repair [10]. Studies have shown that abnormal m^6^A modification is a key mechanism in this pathological process [93,141]. In particular, the dysfunction of the METTL3/METTL14 complex and its regulatory network plays a crucial role [127]. These epigenetic regulatory events disrupt key signaling pathways, disrupting the dynamic balance of muscle regeneration.

In aplastic disorders, the dysfunction of m^6^A modification is first manifested in the abnormality of writers. After the deletion of METTL3, the m^6^A on the transcripts of *Notch1* and *MNK2* decreases, leading to decreased stability and impaired protein synthesis during skeletal muscle regeneration [93,131]. These two dysfunctions together inhibit the proliferation and differentiation of stem cells. In addition, the dysfunction of FTO leads to decreased stability of the mRNA of *MYOD1* and *MYOG*, further impeding differentiation [20,137]. When mice are treated with an FTO-specific inhibitor, rhein, muscle regeneration is significantly delayed [137]. This suggests that FTO is necessary for maintaining the balance of regeneration.

YTHDC1 dysfunction blocks the splicing and nuclear export of transcripts such as *PI4KB*, inhibiting the proliferation of stem cells driven by the PI4K/AKT/mTOR pathway [18,19,110]. Aberrant activation of YTHDF2 leads to over-degradation of targets such as *STK11*, disrupting the AMP-activated protein kinase (AMPK)/mTOR signal balance and compromising protein homeostasis [109]. Members of the IGF2BP family maintain the ERK signal pathway by stabilizing target mRNA such as *FGFR1*, and their dysregulation directly impairs the ability of satellite cells to proliferate and differentiate [111]. The co-regulatory imbalance of these reader proteins can completely disrupt regenerative homeostasis.

In addition, the m^6^A system’s dysregulation indirectly amplifies regenerative damage by interfering with the biogenesis of miRNAs. The METTL3 gene regulates the processing of muscle-specific miRNAs at both the transcriptional and post-transcriptional levels [105]. The YTHDF2 protein promotes the maturation of precursor miRNAs such as pre-miR-378, thereby supporting differentiation [142]. The METTL14 protein is involved in the regulation of differentiation by targeting miR-133b [108]. MiR-24 and miR-122 maintain the regenerative microenvironment by inhibiting the TGF-β/Smad signaling pathway [143]. When the m^6^A modification is abnormal and disrupts the expression network of these miRNAs, fibrosis may be exacerbated and the function of satellite cells may deteriorate further.

Overall, the epitranscriptomic imbalance involving m^6^A is a key issue in regenerative failure of skeletal muscle. The dysfunctional interaction between writers, erasers, and readers can impair the regeneration of skeletal muscle through multiple pathways, including impaired stem cell activation, disrupted differentiation, disrupted protein homeostasis, and the reconfiguration of profibrotic microenvironments [144,145].

### 4.3. Differential Regulation of Myogenic Factors by m^6^A in the Replicative Context

The ‘one-way timer’ in the developmental context emphasizes the irreversibility of direction. m^6^A in skeletal muscle regeneration, on the other hand, reflects more functional programmability. The same modification system can dynamically adjust target priorities and functional outputs at different stages of tissue repair, adapting to the multi-stage transcriptome demands from resting state to proliferation and amplification to final differentiation. In light of this feature, the core characteristics of m^6^A regulation in regeneration can be summarized as the principle of ‘stage and factor specificity’ [127].

Compared to the developmental timer, the m^6^A regulation in regeneration differs in two respects. First, there is the reversibility of the starting point. In development, m^6^A drives a one-way change from an undifferentiated state to a differentiated one. In regeneration, starting from the reactivation of the resting MuSC, the m^6^A system serves the transcriptome transformation of ‘de-resting’ [18]. Thus, the same writer may have different regulatory tasks in early regeneration and late differentiation. There is an extension of the eraser’s function. The eraser is mainly a supportive mechanism to consolidate the direction of differentiation. However, in regeneration, demethylation factors such as FTO and ALKBH5 also reshape the activity of pathways such as p38 MAPK and TGF-β, participating in signal transduction that drives regeneration [99,100]. This suggests that demethylation itself may be part of the regeneration process.

The same m^6^A marker can produce synergistic, antagonistic, or even opposite regulatory effects in different transcripts, at different reader sites, and at different time points. This intrinsic plasticity provides molecular-level flexibility for regenerative efficiency and explains why interventions on single m^6^A regulators often exhibit phenotypic heterogeneity [127]. From a therapeutic perspective, the dynamic programmability of the m^6^A system during regeneration may provide more intervention space than the developmental timer, promoting targeted muscle repair in stages rather than relying on overall reversal [103,137]. 

**Figure 2:**
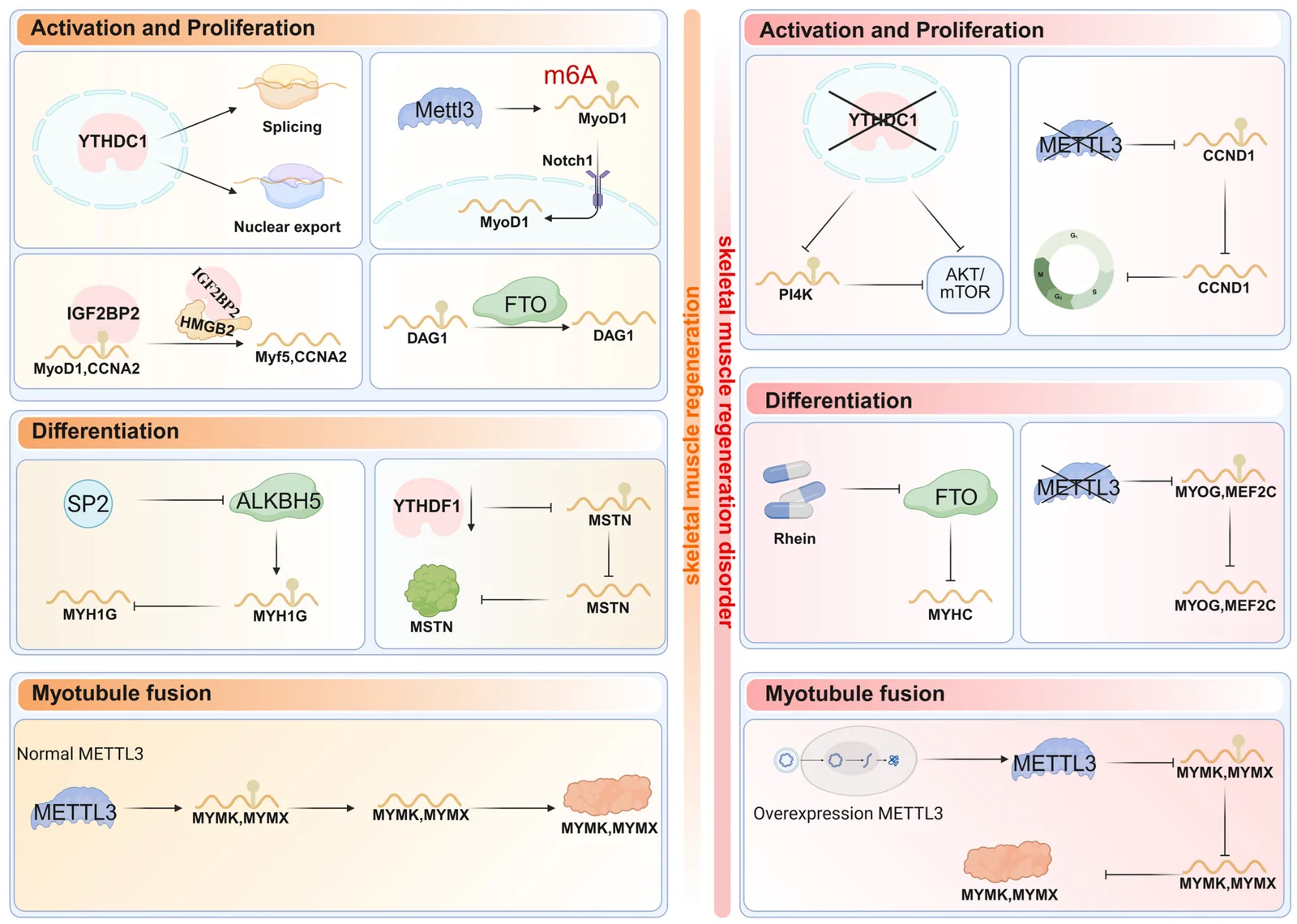
Phase-specific regulation of RNA modification in skeletal muscle regeneration and pathology. Differential regulation of RNA modification enzymes during three phases of myoblast proliferation, differentiation, and fusion in physiological (**left**) and pathological (**right**) states. In physiological states, co-promoter signals such as YTHDC1 and METTL3 promote proliferation, and during differentiation, ALKBH5 and YTHDF1 are de-inhibited. During fusion, METTL3 up-regulates fusion-related factors to form myotubes. In pathological states, the loss or abnormal expression of these modification enzymes (such as METTL3 over-expression) disrupts the proliferation-related pathways (PI4K/AKT/mTOR, CCND1), blocks key differentiation factors (MYOG, MEF2C), and inhibits fusion factors: myomaker, myoblast fusion factor (MYMK), myomixer, myoblast fusion factor (MYMX), ultimately leading to regeneration disorder. The light orange area on the left represents normal skeletal muscle regeneration, while the light purple area on the right indicates regenerative disorders. Each side is further divided into three sections from top to bottom: ‘Activation and Proliferation-Differentiation-Myotubular Fusion’, the ‘→’ indicate positive promotion, the ‘┤’ indicates an inhibitory effect, the ‘X’ represents knockdown. The yellow wavy lines indicating RNA, colored blobs representing proteins, and ‘pill’ representing drug intervention. Abbreviations: cyclin A2 (CCNA2); myosin heavy chain 1G (MYH1G); transcription factor Sp2 (SP2); myostatin (MSTN).

Figure 2 summarizes these findings.

## 5. m^6^A Regulates Skeletal Muscle Atrophy

Muscle atrophy is a key manifestation of skeletal muscle dysfunction, which is commonly caused by various diseases and physiological conditions such as denervation atrophy, cancer-related depletion, aging, and glucocorticoid-induced conditions [116,141,145]. Its molecular mechanisms involve multiple aspects such as imbalances in protein synthesis and degradation, energy metabolism disorders, inflammatory responses, and stem cell dysfunction [146]. In recent years, RNA modification, particularly m^6^A, has been implicated as an important regulator in the occurrence and progression of muscle atrophy [147,148,149]. 

### 5.1. Regulation of RNA Modifications in Different Types of Muscle Atrophy

Different atrophy types exhibit different patterns of m^6^A modification and downstream effects. Table 2 summarizes the m^6^A-related regulatory factors and their downstream effects in five skeletal muscle atrophy models: neurodegenerative atrophy, cancer cachexia atrophy, glucocorticoid-induced atrophy, age-related muscle atrophy, and neuromuscular diseases.

In all models, the m^6^A modification level changes globally, involving three classes of molecules: methyltransferase (METTL3, METTL14), demethylase (FTO, ALKBH5), and recognition proteins (YTHDF2). However, the downstream pathways vary across the models. The ubiquitin-proteasome system (UPS), a conserved protein degradation pathway, is abnormally activated in most atrophy models. However, the key enzymes and upstream signaling axes driving UPS are different in each model, with significant differences in the dominance of methyltransferase and demethylase, as well as the target selection preferences of recognition proteins. The same regulatory factor may contribute to completely different downstream effects in different atrophy models. For example, FTO can antagonize protein degradation in glucocorticoid-induced atrophy, while it is associated with decreased mitochondrial function in age-related muscle atrophy. These differences suggest that the regulation of m^6^A modification for muscle atrophy does not follow a single pattern but exhibits diverse response strategies depending on different pathological stimuli. The next section will discuss the regulatory mechanisms behind these differences in detail.

### 5.2. Regulatory Mechanisms of m^6^A in Muscle Atrophy

RNA modification, particularly m^6^A, plays a central role in skeletal muscle atrophy by regulating the dynamic balance between protein synthesis and degradation [145,149]. RNA sequencing analysis has revealed significant changes in gene expression in atrophy, suggesting that RNA modification may be altered [155].

In protein degradation, imbalances of m^6^A modifications directly drive sarcoplasmic protein loss through abnormal activation of the ubiquitin-proteasome system (UPS). In the model of neurodegenerative atrophy, ALKBH5 removes m^6^A modifications from histone deacetylase 4 (*HDAC4*) mRNA to enable it to escape degradation, subsequently activating the forkhead box O3 (FOXO3) signal pathway and enhancing UPS-mediated protein breakdown [21]. Consistently, global m6A alterations have been observed to govern UPS-mediated protein degradation in denervation atrophy [150]. In cancerous cachexia, exosomes derived from pancreatic cancer cells deliver miR-1247-5p to skeletal muscle cells, which selectively inhibits METTL14, leading to a decrease in overall m^6^A levels and an abnormal increase in the stability of *FOXO3* mRNA, which triggers the F-box protein 32 (*Atrogin-1*)/muscle RING-finger protein 1 (MuRF1) degradation pathway [148]. While exosomal miR-223-5p similarly contributes to muscle wasting, likely through modulation of related targets [156]. YTHDF2 mediates the degradation of ankyrin repeat and SOCS box containing 2 (*ASB2*) mRNA in an m^6^A-dependent manner, inhibiting the TGF-β/SMAD3 signal axis and downstream atrophy gene programs; YTHDF2 deletion leads to over-activation of the proteasome, accumulation of ubiquitinated proteins, and autophagic stress, which directly disrupts muscle protein homeostasis and drives muscular atrophy [157].

On the anabolic side, the disorder of m^6^A modification compromises both protein synthesis and energy supply. Under glucocorticoid exposure, m^6^A modification affects the supply of protein synthesis substrates by regulating the expression of the amino acid transporter SLC7A7 [151]. FTO inhibitor, rhein, significantly delays myoblast differentiation and skeletal muscle regeneration, suggesting that FTO inhibition may exacerbate muscle atrophy [137]. It is suggested that FTO promotes atrophy by inhibiting both anabolic and regenerative pathways. Exercise intervention may counteract atrophy by inhibiting the expression of myostatin, a myostatin-like growth factor mediated by YTHDF1. This suggests that m^6^A modification is also a molecular target of physiological atrophy-resistance adaptations [158].

In neuromuscular diseases, m^6^A dysregulation exhibits distinct disease specificity. In C9orf72-related amyotrophic lateral sclerosis (ALS) and frontotemporal dementia (FTD), a global m^6^A reduction disrupts RNA metabolism and promotes neurodegeneration [159]. Changes in m^6^A levels also affect the RNA-binding properties and pathological aggregation of TAR DNA-binding protein 43 (*TDP-43*) [160]. Abnormalities of DNA and RNA methylation are detected simultaneously in superoxide dismutase 1 (SOD1) mouse models and ALS patients, suggesting that epigenetic disorders are a common pathologic feature of neuromuscular diseases [154]. Conversely, up-regulation of METTL3 in facioscapulohumeral muscular dystrophy (FSHD) myoblasts leads to excessive m^6^A modification, which in turn inhibits differentiation and hinders muscle repair [153]. This opposite m^6^A pathology suggests that it is the shift in modification homeostasis rather than a simple increase or decrease in modification levels that is critical.

In addition, m^6^A can synergistically regulate muscle atrophy with miRNAs. miR-1247-5p regulates m^6^A levels by targeting METTL14, which affects the FOXO3 signal pathway and promotes cancer-related muscle atrophy [148]. miRNAs such as miR-29a and miR-206 are also involved in regulating muscle atrophy [161,162]. The biogenesis and function of these miRNAs may be directly regulated by m^6^A modifications, forming a positive feedback regulatory loop between epigenomes and non-coding RNAs.

The above findings reveal that the epitranscriptome, with m^6^A as the core, is unbalanced. This imbalance is driven by multiple pathways, including increased protein degradation, impaired anabolism, mitochondrial dysfunction, and reshaping of the inflammatory microenvironment, which leads to the development of muscular atrophy [141,145]. This provides a clear molecular framework for developing precise intervention strategies targeting specific modification enzymes and their key substrates.

### 5.3. Regulatory Mechanisms of m^6^A in Other Skeletal Muscle Disorders

In addition to muscle atrophy, RNA modification is involved in a variety of skeletal muscle pathologies, including senile sarcopenia, genetic myopathies, inflammatory injuries, and metabolic muscle diseases. Ultrastructural evidence suggests that both patients with myotonic dystrophy and sarcopenia exhibit significantly impaired RNA transcription and maturation processes in skeletal muscles [163]. This suggests that impaired RNA metabolism is a common pathological feature across muscle diseases with different etiologies, and the dysregulation of epigenetic modification may be an important molecular basis for this commonality [10,25].

With age, skeletal muscle mass and function gradually decline, leading to sarcopenia and age-related muscle dysfunction. Age-dependent changes in m^6^A modification are an important molecular basis for muscle function decline. The epigenomic map of senescent tissues in primates shows systematic reprogramming of m^6^A modification with age. The expression and activity of core enzymes such as METTL3 and FTO in skeletal muscle directly affect the differentiation potential, energy metabolism, and antioxidant stress of muscle cells [152,164]. Mutations of METTL3 that stabilize *NPNT* mRNA with m^6^A modification dependence maintain myotube homeostasis; deletion of METTL3 leads to myotube atrophy, apoptosis, and accelerated senescence [152]. m^6^A modification also participates in the regulation of age-related signaling pathways such as FOXO3/HDAC4 by regulating muscle-specific miRNA and lncRNA [16]. Endurance exercise has been shown to counteract senile muscle remodeling by inhibiting YTHDF1-mediated myostatin expression [158,165]. This suggests that the m^6^A system is both a pathological mediator of senescence and a plastic target of exercise intervention.

Abnormal RNA modification is considered an important molecular basis of inherited myopathies. m^6^A modification affects the stability and function of muscle cells by regulating the expression of muscle structural proteins, signal molecules, and related non-coding RNAs [25,166]. Dysregulation of m^6^A-related proteins such as METTL3 and YTHDF2 leads to an imbalance between protein synthesis and degradation, and abnormal cellular stress responses [92,157,167]. These factors also contribute to the pathological progression of muscular dystrophy by affecting autophagy, apoptosis, and inflammatory responses [10,25]. lncRNAs play an extensive regulatory role in skeletal muscle development and muscle diseases, and the dysregulation of m^6^A-related factors can directly disrupt the expression and interaction networks of these lncRNAs, further impairing the regeneration and repair of diseased muscles [168].

In muscle inflammation and injury, m^6^A modification regulates the stability and translation efficiency of mRNA of inflammation-related genes and signal pathways (such as nuclear factor kappa-light-chain-enhancer of activated B cells (NF-κB) and MAPK), influencing the intensity and duration of inflammatory responses. It also participates in post-inflammatory structural remodeling and functional recovery through miRNA and lncRNA networks [10,16,25].

Furthermore, RNA modification plays an important role in abnormal muscle metabolism and related metabolic diseases such as obesity, insulin resistance and mitochondrial myopathy. FTO regulates mitochondrial function and muscle fiber remodeling via the DHA/m^6^A/DDIT4/PGC-1α signal axis [117,126]. FTO with the obesity-risk allele can accelerate insulin resistance in human skeletal muscle cells [169].

Overall, the regulatory functions of RNA modification span the entire spectrum of skeletal muscle diseases, from senescence and degeneration, genetic defects, inflammatory injury, and metabolic disorders [166,167]. Different modification types shape disease phenotypes through distinct enzyme-substrate combinations in their respective pathological contexts, providing a broad range of molecular targets for precise epigenomic interventions for specific types of myopathies.

### 5.4. De-Myogenic Shift in m^6^A Regulatory Targets in the Atrophic Context

When m^6^A regulation shifts from physiological to pathological microenvironments, the targeting logic changes significantly. In muscle atrophy, rather than an overall increase or decrease in activity, the m^6^A modification system exhibits a ‘de-myogenic’ bias: epigenetic regulatory resources no longer support muscle-derived processes (*MYOD1*, *MYOG*, etc.), but instead support pathological processes of catabolism, inflammation amplification, and fibrotic remodeling [21,150].

Disease signals, such as inflammatory factors, glucocorticoids, and tumor-derived exosomes, alter the targeting selectivity of the writer-reader-eraser. m^6^A modifications, which had been concentrated on pro-differentiation transcripts, enhance the FOXO3/Atrogin-1 catabolic axis and related stress pathways instead [151,156]. This is more a competitive redistribution of modification resources than a simple change in enzyme activity. This shift does not occur in isolation. It is associated with aging-related epigenetic drift, chronic inflammation, and oxidative stress signals, and may form a positive feedback loop, causing the epigenome to shift more rapidly from muscle maintenance to pathological remodeling [129].

In the atrophy state, target selectivity is redirected by pathological signals, and the system becomes an ‘adapter’ of alienated metabolism. Therefore, simply restoring the activity of a certain modifier enzyme may have limited effects. Redirecting m^6^A modification resources to myogenic targets and reversing the ‘de-myogenic’ target shift could be important directions for intervening in muscle atrophy [141,145]. 

**Figure 3:**
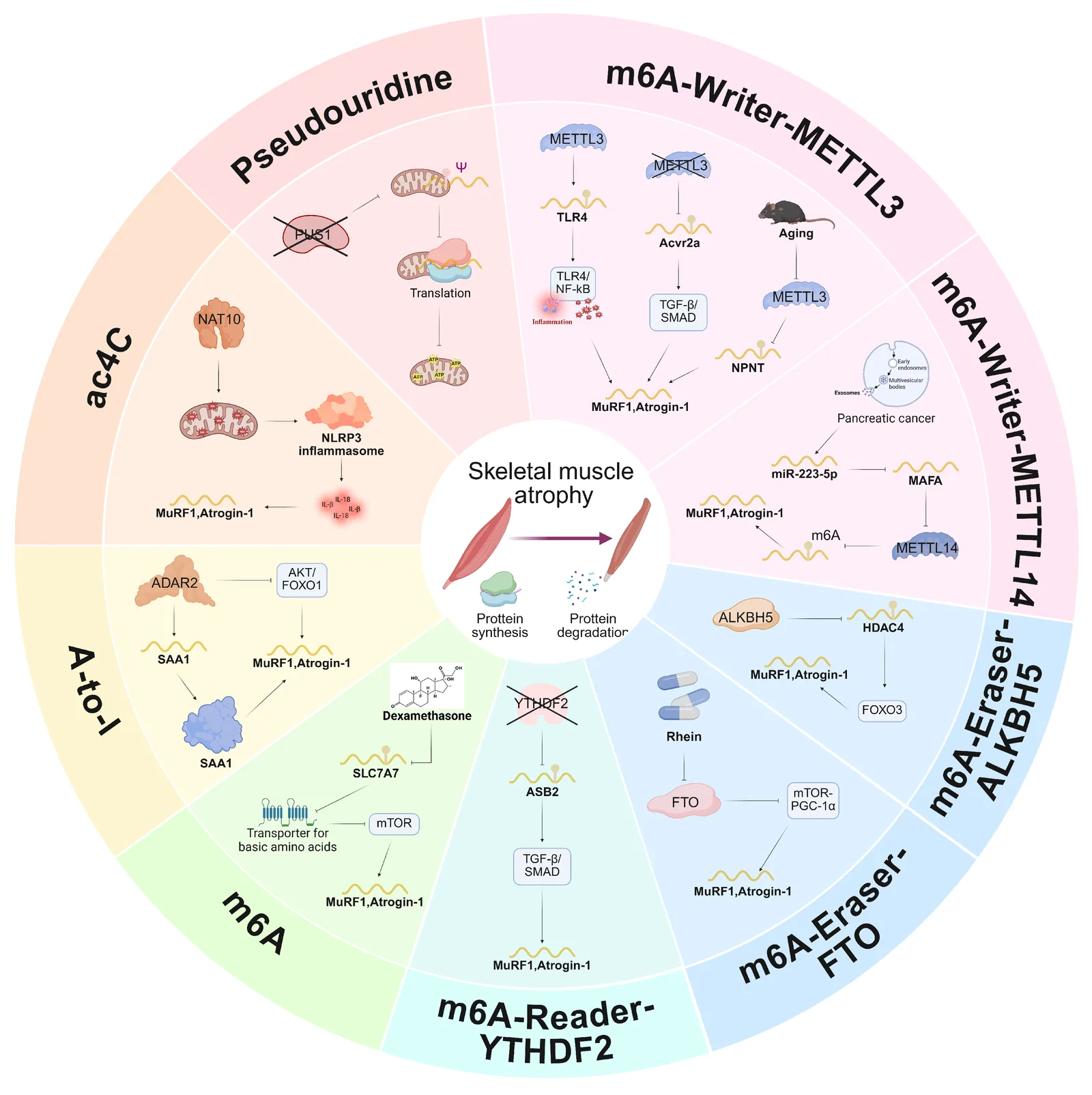
RNA modifications and their regulatory factors in skeletal muscle atrophy. Atrophy is driven by an imbalance between decreased protein synthesis and increased degradation, which ultimately leads to the up-regulation of atrophy markers MuRF1 and Atrogin-1. m^6^A modification promotes protein degradation or inhibits synthesis through TGF-β/SMAD, ALKBH5, FTO, and YTHDF2, among other signaling axes, such as METTL3/METTL14, FTO, mTOR/PGC-1α, etc. Pseudo-uridine modification, A-to-I editing (ADAR2/AKT1/ forkhead box O1 (FOXO1)), and NAT10/NLRP3 inflammasome are also involved in the atrophy process through different upstream pathways, all of which ultimately converge on the up-regulation of MuRF1 and Atrogin-1. The colors are divided by modification type, with flesh pink indicating pseudouridinase, pale pink indicating m^6^A writer enzyme Mettl3/14, blue indicating m^6^A eraser/reader ALKBH5, FTO, and YTHDF2, light yellow indicating A-to-I editor ADAR2, light orange indicating ac4C, and light green indicating m^6^A metabolic regulation. the ‘→’ indicate positive promotion, the ‘┤’ indicates an inhibitory effect, the ‘X’ represents knockdown The yellow dashed line represents RNA, the colored blob represents protein, and the drug represents drug intervention. Abbreviations: MAF bzip transcription factor A (MAFA); serum amyloid A1 (SAA1); Toll-like receptor 4 (TLR4).

Figure 3 summarizes these findings, and also depicts the mechanisms by which A-to-I editing, pseudouridylation, and ac4C regulate skeletal muscle.

## 6. Regulation of Skeletal Muscle by Other RNA Modifications

Development of skeletal muscle is co-regulated by multiple RNA modifications. m^5^C and A-to-I editing are also important in skeletal muscle proliferation, differentiation, and myotube formation. During the proliferative phase, m^5^C modification is dynamic, promoting myoblast proliferation by regulating the expression of proliferation-related genes [170]. After entering the differentiation phase, m^5^C modifications on ribosomal RNA ensure proper ribosome assembly and translational support [171]. ALYREF recognizes mRNA such as Y-box binding protein 2 (YBX2) and smoothened (SMO) in an m^5^C-dependent manner, facilitating their nuclear export and promoting the differentiation process [22]. Global reshaping also occurs in A-to-I editing mediated by the ADAR (Adenosine Deaminase Acting on RNA) family. These edits alter RNA sequences, regulate myocyte-specific variable splicing, and control selenoprotein expression by termination codon recoding to maintain redox homeostasis [172,173]. Editing events and splicing regulation are closely linked to the determination of muscle fiber type differentiation and metabolic adaptations [171,173].

In terms of regeneration and metabolism, the m^5^C modification mediated by NSUN2-ALYREF ensures efficient expression of muscle-forming genes and supports the metabolic needs of the regeneration process [22]. A-to-I dysregulation interferes with selenoprotein codon recoding, impairs redox balance, and hinders myotube formation [172,173].

The pathogenesis of skeletal muscle atrophy also includes additional types of RNA modifications. Mutations in PUS1 (pseudouridine synthase 1) result in a defect in tRNA pseudouridine (Ψ) modification, impairing mitochondrial translation and triggering a syndrome characterized by myasthenia, known as myopathy, lactic acidosis, and sideroblastic anemia (MLASA) syndrome [174,175]. In inflammation-driven atrophy, inhibiting NAT10 can reduce mitochondrial ROS production, block NLRP3 inflammatory activation, and exert a protective effect [176]. ADAR2-catalyzed A-to-I editing up-regulates SAA1 expression, exacerbating non-alcoholic fatty liver disease and its associated skeletal muscle atrophy, extending the regulatory scope to muscle atrophy associated with metabolic stress [177]. These multiple pathways integrate to maintain a dynamic balance during skeletal muscle development, regeneration, and atrophy.

## 7. Interactions Between RNA and Other Epigenetic Modifications

The development and regeneration of skeletal muscle are regulated not by a single epigenetic mechanism, but by a highly integrated multi-dimensional epigenetic regulatory system [33,145,178]. At the heart of this system is two-way molecular feedback between DNA demethylation and RNA m^6^A modification. DNA methylation occurs predominantly at cytosine-phosphate-guanine (CpG) sites, and methylation of promoter regions typically leads to gene silencing, while Ten-eleven Translocation (TET) proteins mediate active demethylation [179]. During skeletal muscle development, demethylation of promoters of myogenic genes (e.g., *MYOD1*, *MYOG*) initiates the differentiation program in myoblasts [180]. Ten-eleven Translocation 1 (TET1)-mediated DNA demethylation maintains the transcription of METTL3, and the m^6^A tags catalyzed by METTL3 stabilize the TET1 transcript via YTHDF2, forming a self-reinforcing loop that provides epigenetic accessibility to myogenic loci [181]. Chromatin-related regulatory RNA (CARRNA) carrying m^6^A modification can also recruit TET1 to specific genomic sites, driving local DNA demethylation [182]. Simultaneously, m^6^A modification on CARRNA can promote its own degradation through YTHDC1, controlling its abundance on the chromatin; once m^6^A is depleted, CARRNA accumulates, opening the chromatin and activating transcription [183]. Histone H3 trimethylated at lysine 36 (H3K36me3) histone modification guides m^6^A deposition onto nascent RNAs, and m^6^A is responsible for removing suppressive tags such as Histone H3 dimethylated at lysine 9 (H3K9me2) [184,185]. Chromatin state and RNA modification are bidirectionally connected through these mechanisms.

In this multi-layered regulatory system, non-coding RNAs act as both regulatory elements and functional scaffolds. Specific long non-coding RNAs, such as Dum and lncMREF, anchor DNA methyltransferases or adenosine triphosphate (ATP)-dependent chromatin remodeling complexes to target genomic regions [186,187]. At the same time, long non-coding RNAs, circRNAs and a large number of miRNAs are also involved, performing sophisticated post-transcriptional regulation [188,189,190,191,192,193,194,195]. During skeletal muscle development, long non-coding RNAs (lncRNAs) are themselves widespread substrates for m^6^A modification. This dual role places lncRNAs at the intersection of the epigenome and the chromatin landscape, making them critical junctions [86].

DNA demethylation, m^6^A modification, histone modifications, and non-coding RNAs interact through multiple positive feedback loops and transcriptional couplings, shattering traditional boundaries between epigenetic layers. Systematically deciphering the causal hierarchy and in vivo temporal and spatial dynamics of this self-consistent network has the potential to fundamentally alter our understanding of muscle formation mechanisms and provide a more complete epigenetic therapeutic framework for degenerative diseases such as muscular dystrophy and sarcopenia.

## 8. The Role and Future Applications of RNA Modifications in Skeletal Muscle

m^6^A is a widely studied RNA modification in skeletal muscle development, regeneration, and atrophy. It regulates key transcription factors, non-coding RNAs, and signaling pathways to form a multi-layered molecular network that determines the fate of skeletal muscles. Enzymes such as METTL3/14, FTO, and ALKBH5 have different functions in different microenvironments. They not only participate in muscle cell differentiation and regeneration but also mediate protein degradation and metabolic disorders in disease states. Additionally, m^6^A interacts with non-coding RNAs such as miRNAs, lncRNAs, and circRNAs to jointly regulate skeletal muscle development.

In terms of treatment, the progress of RNA modification targeted intervention in different skeletal muscle diseases is not uniform. There has been more research on muscular atrophy and cachexia. In neurodegenerative muscular atrophy, the imbalance of ALKBH5-HDAC4-FOXO3 axis and m^6^A drives the activation of the ubiquitin-proteasome system. In pancreatic cancer cachexia, miRNAs carried by tumor-derived small extracellular vesicles inhibit METTL14 and enhance FOXO3 signaling. Sepsis-induced muscular atrophy involves the regulation of the ROS/NLRP3 pathway by the ac4C-modifying enzyme NAT10. In sarcopenia, m^6^A reprogramming is associated with primate aging and decreased muscle function, but interventions are still primarily based on physiological adjustments such as exercise and nutrition. In muscular dystrophies such as FSHD, the levels of METTL3 and m^6^A are elevated, but evidence for targeted interventions is still limited. FTO inhibitors, oligonucleotides, and gene editing have shown potential in animal models but face three problems in clinical translation. First, the function of RNA modification is context-dependent: METTL3 can promote the regeneration of muscle stem cells but can also be abnormally elevated in some muscle diseases. Second, the deep coupling of RNA modification to non-coding RNA networks makes it difficult to reshape complex pathological circuits with a single molecular target. Third, cellular heterogeneity and temporal dynamics increase the difficulty of accurate delivery and may disrupt the epigenome homeostasis of other tissues. Overall, RNA modification offers a new intervention direction for skeletal muscle diseases. However, to achieve clinical translation, disease stratification, spatiotemporal analysis, and safer delivery methods are needed. 

**Figure 4:**
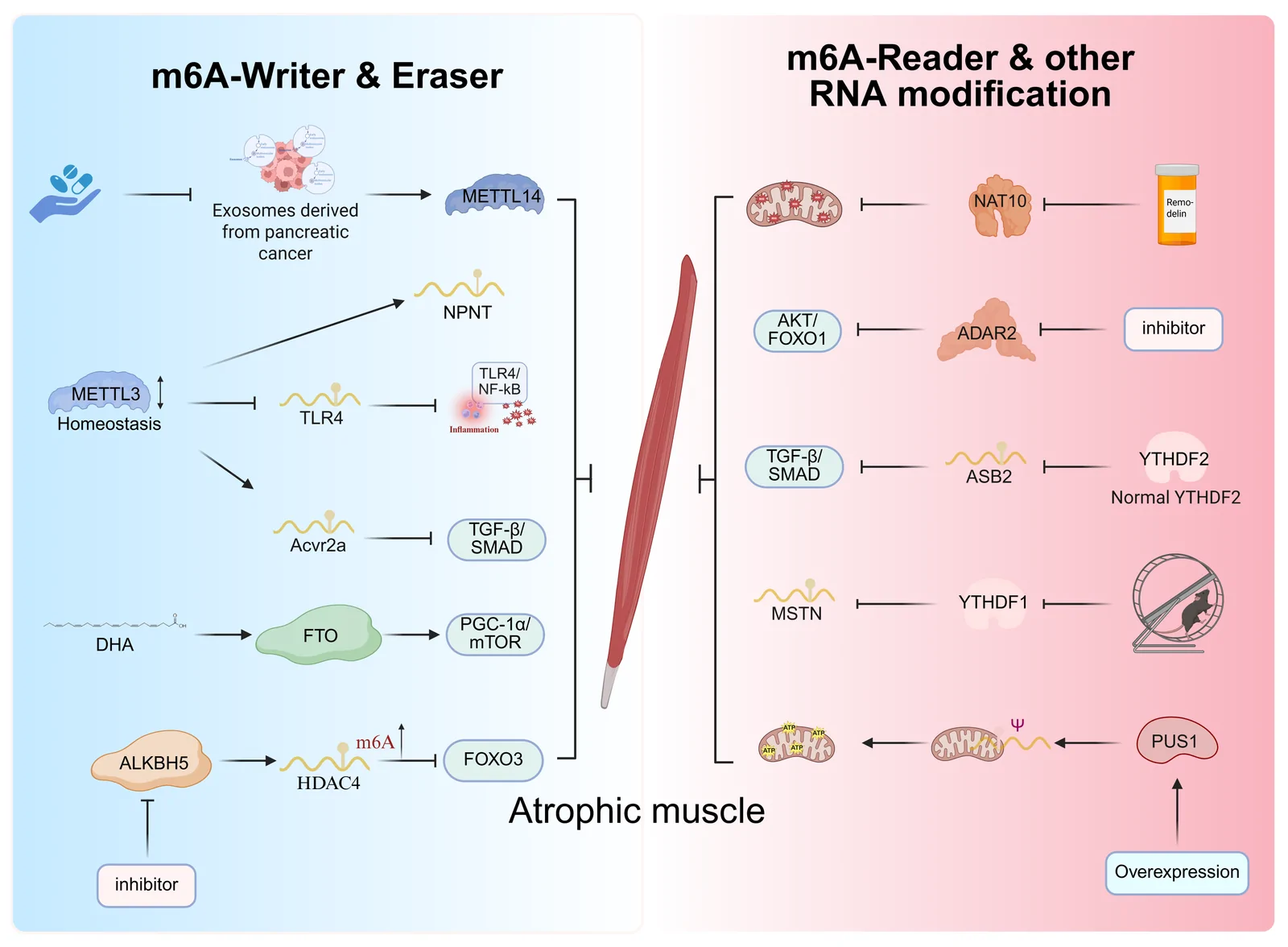
Strategies and molecular mechanisms for muscle atrophy treatment. Intervention strategies for these modification pathways include multiple modification types: inhibiting m^6^A erase enzymes FTO or ALKBH5 to regulate the synthesis/catabolism axis, blocking NAT10 to improve mitochondrial function and inhibit inflammation, interfering with A-to-I editing enzyme ADAR2 to correct AKT1/FOXO1 signaling, targeting reading proteins YTHDF2 and YTHDF1 to intervene in TGF-β/SMAD pathways and MSTN respectively, and promoting pseudouridination by overexpressing PUS1 to enhance mitochondrial tRNA function. The ‘→’ indicate positive promotion, the ‘┤’ labeled ‘Inhibition’ indicates suppression, the ‘↔’ indicates two-way regulation. The yellow wavy lines indicating RNA, colored blobs representing proteins, and ‘pill’ representing drug intervention.

Figure 4 summarizes these findings.

Currently, most research focuses on RNA types such as mRNA, miRNA, lncRNA, and circRNA. The regulatory role of RNA modification on other non-coding RNAs such as tRNA and rRNA remains unclear, and their biological significance needs further exploration. Additionally, existing research mostly focuses on m^6^A, m^5^C, and A-to-I editing, and the exploration of other RNA modifications is still in its early stages. The spatio-temporal specificity and coordinating mechanisms of RNA modifications in skeletal muscle need to be systematically clarified. In the future, the cross-integration of emerging technologies such as single-cell multi-omics, spatial transcriptomics, and direct RNA sequencing could reveal the dynamic picture of multiple modifications in different RNA types in development, adaptation, and diseases. Further expanding the research on RNA modifications in skeletal muscle and deepening the understanding of the epigenetic transcriptional regulatory network in skeletal muscle could also provide new theoretical bases and molecular targets for early diagnosis, biomarker screening, and precise treatment of related diseases.

## Figures and Tables

**Table 1:** Summary of common RNA modification types and their modifying enzyme systems.

RNA Modification Class	Representative Modification	Main Distribution	Writer	Reader	Eraser	Reference
Methylation	m^6^A	mRNA, lncRNA	METTL3/14, WTAP	YTHDF1-3, IGF2BPs	FTO, ALKBH5	[34]
	m5C	mRNA, tRNA, rRNA	NSUN2/4, DNMT2	ALYREF, YBX1	TETs, ALKBH1	[35]
	m^1^A	tRNA (Pos 58), mRNA	TRMT6/61A	Unknown	ALKBH1, ALKBH3	[31]
	m^7^G	mRNA, tRNA	METTL1/WDR4	Unknown	Unknown	[32]
	m^6^Am	mRNA Cap-adjacent	PCIF1	Unknown	FTO	[36]
	m^6^,^6^A	18S rRNA	DIMT1	Unknown	Unknown	[37]
	m^3^C	tRNA, mRNA	METTL8	Unknown	ALKBH1	[38]
	m^1^G	tRNA, rRNA	TRMT5, TRMT10A	Unknown	ALKBH1	[39]
	m^2^G	tRNA	TRMT11	Unknown	Unknown	[40]
	m^22^G	tRNA, rRNA	TRMT1	Unknown	Unknown	[41]
	m^5^U	tRNA, rRNA	TRMT2A	Unknown	Unknown	[42]
	Nm	rRNA, mRNA, snRNA, snoRNA	Fibrillarin (FBL)	Unknown	Unknown	[43]
	m^4^C	12S rRNA, tRNA	METTL15	Unknown	Unknown	[44]
Pseudouridylation	Ψ	rRNA, tRNA, mRNA	PUS enzymes, DKC1	Unknown	Unknown	[45]
Deamination/Editing	I	dsRNA (Alu repeats)	ADAR1, ADAR2	Unknown	Unknown	[46]
	C-to-U	mRNA	APOBEC3A	Unknown	Unknown	[47]
Dihydrouridylation	D	tRNA (D-loop)	DUS enzymes	Unknown	Unknown	[48]
Thiolation	s^2^U	tRNA (Wobble)	CTU1, CTU2	Unknown	Unknown	[49]
	τm^5^s^2^U	Mitochondrial tRNA	MTO1, TRMU	Unknown	Unknown	[50]
	mcm^5^s^2^U	tRNA (Wobble)	ELP3 (Elongator)	Unknown	Unknown	[51]
Acetylation	ac^4^C	mRNA, rRNA	NAT10	Unknown	Unknown	[52]
Complex side-chain modification	t^6^A	tRNA (Pos 37)	KEOPS Complex	Unknown	Unknown	[53]
	Q	tRNA (Wobble)	QTRT1/2	Unknown	Unknown	[54]
	yW	tRNA-Phe	TYW1-4	Unknown	Unknown	[55]
	acp^3^U	tRNA, rRNA	DTWD1/2	Unknown	Unknown	[56]
	mcm^5^U	tRNA	ALKBH8	Unknown	Unknown	[57]
	ms^2^t^6^A	tRNA	CDK5RAP1	Unknown	Unknown	[58]
Non-canonical/Alternative caps	TMG	snRNA	TGS1	Snurportin1	Unknown	[59]
	NAD+ cap	5′ end of mRNA	Pol II	Unknown	DXO	[60]
Glycosylation	GlycoRNA	Small RNAs	Unknown	Siglecs	Unknown	[61]

Note: m^6^A, m^5^C, m1A, Ψ, and I are found in skeletal muscle. NAT10, the ac4C writer, is associated with muscle disease; yet ac4C function in muscle remains unclear. Abbreviations: insulin-like growth factor 2 mRNA-binding proteins (IGF2BPs); fat mass and obesity-associated protein (FTO); NOP2/Sun RNA methyltransferase family member 4 (NSUN4); DNA (cytosine-5)-methyltransferase-like protein 2 (DNMT2); Y-box binding protein 1 (YBX1); ten-eleven translocation proteins (TETs); alkB homolog 1, histone H2A dioxygenase (ALKBH1); tRNA methyltransferase 6 (TRMT6); tRNA methyltransferase 61A (TRMT61A); alkB homolog 3, alpha-ketoglutarate-dependent dioxygenase (ALKBH3); methyltransferase-like 1 (METTL1); WD repeat domain 4 (WDR4); N^6^,2′-O-dimethyladenosine (m^6^Am); phosphorylated CTD-interacting factor 1 (PCIF1); eukaryotic translation initiation factor 4F (eIF4F); N^6^,N^6^-dimethyladenosine (m^6^,^6^A); DIM1 rRNA methyltransferase and ribosome maturation factor (DIMT1); 3-methylcytidine (m^3^C); methyltransferase-like 8 (METTL8); N^1^-methylguanosine (m^1^G); tRNA methyltransferase 5 (TRMT5); tRNA methyltransferase 10A (TRMT10A); N^2^-methylguanosine (m^2^G); tRNA methyltransferase 11 (TRMT11); N^2^,N^2^-dimethylguanosine (m^22^G); tRNA methyltransferase 1 (TRMT1); 5-methyluridine (m^5^U); tRNA methyltransferase 2A (TRMT2A); 2′-O-methylation (Nm; 2′-O-methylribose); small nuclear RNA (snRNA); small nucleolar RNA (snoRNA); fibrillarin (FBL); N^4^-methylcytidine (m^4^C); methyltransferase-like 15 (METTL15); pseudouridine synthase enzymes (PUS enzymes); dyskerin pseudouridine synthase 1 (DKC1); inosine (I); double-stranded RNA (dsRNA); adenosine deaminase acting on RNA 2 (ADAR2); cytidine-to-uridine editing (C-to-U); apolipoprotein B mRNA editing enzyme catalytic subunit 3A (APOBEC3A); dihydrouridine (D); dihydrouridine synthase enzymes (DUS enzymes); 2-thiouridine (s^2^U); cytosolic thiouridylase subunit 1 (CTU1); cytosolic thiouridylase subunit 2 (CTU2); 5-taurinomethyl-2-thiouridine (τm^5^s^2^U); mitochondrial tRNA translation optimization 1 (MTO1); tRNA 5-methylaminomethyl-2-thiouridylate methyltransferase (TRMU); 5-methoxycarbonylmethyl-2-thiouridine (mcm^5^s^2^U); elongator complex protein 3 (ELP3); N^4^-acetylcytidine (ac^4^C); N-acetyltransferase 10 (NAT10); N^6^-threonylcarbamoyladenosine (t^6^A); kinase, endopeptidase and other proteins of small size complex (KEOPS complex); queuosine (Q); queuine tRNA-ribosyltransferase catalytic subunit 1 (QTRT1); queuine tRNA-ribosyltransferase auxiliary subunit 2 (QTRT2); wybutosine (yW); tRNA-yW synthesizing protein 1 (TYW1); tRNA-yW synthesizing protein 2 (TYW2); tRNA-yW synthesizing protein 3 (TYW3); tRNA-yW synthesizing protein 4 (TYW4); 3-(3-amino-3-carboxypropyl)uridine (acp^3^U); DTW domain containing 1 (DTWD1); DTW domain containing 2 (DTWD2); 5-methoxycarbonylmethyluridine (mcm^5^U); alkB homolog 8, tRNA methyltransferase (ALKBH8); 2-methylthio-N^6^-threonylcarbamoyladenosine (ms^2^t^6^A); CDK5 regulatory subunit associated protein 1 (CDK5RAP1); trimethylguanosine (TMG); trimethylguanosine synthase 1 (TGS1); nicotinamide adenine dinucleotide cap (NAD^+^ cap); RNA polymerase II (Pol II); decapping exoribonuclease (DXO); glycosylated RNA (GlycoRNA).

**Table 2:** Regulation of m^6^A in different types of muscle atrophy.

Type of Muscle Atrophy	Key Regulators	Downstream Effects	References
Denervation atrophy	ALKBH5, *FOXO3*	UPS-mediated protein degradation	[21,150]
cancer cachexia atrophy	METTL14, *FOXO3*, miR-1247-5P	Activation of the UPS degradation pathway	[148]
Glucocorticoid-induced atrophy	METTL3, *SLC7A7*	Antagonizing atrophy; altered amino acid transport	[151]
Age-related muscle atrophy	METTL3, IGF2BP1, *NPNT*	Musculotubular Degeneration	[152]
Neuromuscular Diseases	METTL3	Neurodegeneration-induced muscle weakness	[153,154]

Note: Abbreviations: forkhead box O3 (*FOXO3*); solute carrier family 7 member 7 (*SLC7A7);* nephronectin (*NPNT*).

## Data Availability

No new data were created or analyzed in this study. Data sharing is not applicable to this article.

## Abbreviations

The following abbreviations are used in this manuscript:

ac^4^C	N^4^-acetylcytidine
acp^3^U	3-(3-amino-3-carboxypropyl)uridine
ACVR2A	Activin A receptor type 2A
ADAR1	Adenosine deaminase acting on RNA 1
ADAR2	Adenosine deaminase acting on RNA 2
AKT	Protein kinase B (PKB)
ALKBH1	Alkb homolog 1, histone H2A dioxygenase
ALKBH3	Alkb homolog 3, alpha-ketoglutarate-dependent dioxygenase
ALKBH5	Alkb homolog 5, RNA demethylase
ALKBH8	Alkb homolog 8, trna methyltransferase
ALS	Amyotrophic lateral sclerosis
ALYREF	Aly/REF export factor
AMPK	AMP-activated protein kinase
APOBEC1-YTH	APOBEC1 cytidine deaminase fused with YTH m^6^A binding domain
APOBEC3A	Apolipoprotein B mrna editing enzyme catalytic subunit 3A
ASB2	Ankyrin repeat and SOCS box containing 2
A-to-I	Adenosine-to-inosine
ATP	Adenosine triphosphate
Atrogin-1	Muscle atrophy F-box
CAMK2B	Calcium/calmodulin-dependent protein kinase II beta
CCNA2	Cyclin A2
CCND1	Cyclin D1
CDK5RAP1	CDK5 regulatory subunit associated protein 1
circRNA	Circular RNA
CpG	Cytosine-phosphate-guanine
C-to-U	Cytidine-to-uridine editing
CTU1	Cytosolic thiouridylase subunit 1
CTU2	Cytosolic thiouridylase subunit 2
D	Dihydrouridine
DAG1	Dystroglycan 1
DART-seq	Droplet-assisted RNA targeting by single-cell sequencing
DDIT4	DNA damage inducible transcript 4
DHA	Docosahexaenoic acid
DIMT1	DIM1 rrna methyltransferase and ribosome maturation factor
DKC1	Dyskerin pseudouridine synthase 1
DNA	Deoxyribonucleic acid
DNMT2	DNA (cytosine-5)-methyltransferase-like protein 2
dsRNA	Double-stranded RNA
DTWD1	DTW domain containing 1
DTWD2	DTW domain containing 2
DUS enzymes	Dihydrouridine synthase enzymes
DXO	Decapping exoribonuclease
eIF4F	Eukaryotic translation initiation factor 4F
eIF4G	Eukaryotic translation initiation factor 4G
ELP3	Elongator complex protein 3
ERK	Extracellular signal-regulated kinase
FAPs	Fibro-adipogenic progenitors
FBL	Fibrillarin
FGFR1	Fibroblast growth factor receptor 1
FOXO1	Forkhead box O1
FOXO3	Forkhead box O3
FSHD	Facioscapulohumeral muscular dystrophy
FTD	Frontotemporal dementia
FTO	Fat mass and obesity-associated protein
GADD45B	Growth arrest and DNA damage-inducible beta
GHR	Growth hormone receptor
GLORI	Glyoxal and nitrite-mediated deamination of unmethylated adenosines
GPCPD1	Glycerophosphocholine phosphodiesterase 1
H3K36me3	Trimethylation of histone H3 at lysine 36
H3K9me2	Dimethylation of histone H3 at lysine 9
HDAC4	Histone deacetylase 4
HMGB2	High mobility group box 2
HNRNPA2B1	Heterogeneous nuclear ribonucleoprotein A2/B1
I	Inosine
IGF1R	Insulin-like growth factor 1 receptor
IGF2BP1	Insulin-like growth factor 2 mrna-binding protein 1
IGF2BP2	Insulin-like growth factor 2 mrna-binding protein 2
IGF2BP3	Insulin-like growth factor 2 mrna-binding protein 3
IGF2BPs	Insulin-like growth factor 2 mrna-binding proteins
KEOPS complex	Kinase, endopeptidase and other proteins of small size complex
LIN28A	Lin-28 homolog A
lncRNA	Long non-coding RNA
m^1^A	N^1^-methyladenosine
m^1^G	N^1^-methylguanosine
m^22^G	N^2^,N^2^-dimethylguanosine
m^2^G	N^2^-methylguanosine
m^3^C	3-methylcytidine
m^4^C	N^4^-methylcytidine
m^5^C	5-methylcytidine
m^5^U	5-methyluridine
m^6^,^6^A	N^6^,N^6^-dimethyladenosine
m^6^A	N^6^-methyladenosine
m^6^Am	N^6^,2′-O-dimethyladenosine
m^7^G	N^7^-methylguanosine
MAFA	MAF bzip transcription factor A
MAPK	Mitogen-activated protein kinase
mcm^5^s^2^U	5-methoxycarbonylmethyl-2-thiouridine
mcm^5^U	5-methoxycarbonylmethyluridine
MEF2C	Myocyte enhancer factor 2C
MeRIP-seq	Methylated RNA immunoprecipitation sequencing
METTL1	Methyltransferase-like 1
METTL14	Methyltransferase-like 14
METTL15	Methyltransferase-like 15
METTL3	Methyltransferase-like 3
METTL8	Methyltransferase-like 8
miRNA	Micro RNA
MLASA	Mitochondrial myopathy, lactic acidosis, and sideroblastic anemia
MNK2	MAPK-interacting kinase 2
MPCs	Myogenic progenitor cells
MRF4	Muscle regulatory factor 4
mRNA	Messenger RNA
ms^2^t^6^A	2-methylthio-N^6^-threonylcarbamoyladenosine
MSTN	Myostatin
MTO1	Mitochondrial trna translation optimization 1
mTOR	Mechanistic target of rapamycin
MuRF1	Muscle RING-finger protein 1
MuSCs	Muscle satellite cells
MYF5	Myogenic factor 5
MYH	Myosin heavy chain
MYH1	Myosin heavy chain 1
MYH1G	Myosin heavy chain 1G, skeletal muscle
MYH7	Myosin heavy chain 7
MYMK	Myomaker, myoblast fusion factor
MYMX	Myomixer, myoblast fusion factor
MYOD1	Myoblast determination protein 1
MYOG	Myogenin
NAD^+^ cap	Nicotinamide adenine dinucleotide cap
NAT10	N-acetyltransferase 10
NEK1	NIMA-related kinase 1
NFATC1	Nuclear factor of activated T-cells
NF-κB	Nuclear factor kappa B
NGS	Next-generation sequencing
NLRP3	NLR family pyrin domain containing 3
Nm	2′-O-methylation (2′-O-methylribose)
Notch	Notch receptor
NPNT	Nephronectin
NSUN2	NOP2/Sun RNA methyltransferase 2
NSUN4	NOP2/Sun RNA methyltransferase family member 4
PAX3	Paired box 3
PAX7	Paired box 7
PCIF1	Phosphorylated CTD-interacting factor 1
PGC-1α	Peroxisome proliferator-activated receptor gamma coactivator 1-alpha
PHKB	Phosphorylase kinase regulatory subunit beta
PI3K	Phosphoinositide 3-kinase
PI4K	Phosphatidylinositol 4-kinase
PI4KB	Phosphatidylinositol 4-kinase beta
PICALM	Phosphatidylinositol binding clathrin assembly protein
Pol II	RNA polymerase II
pre-miRNA	Precursor microrna
PUS enzymes	Pseudouridine synthase enzymes
PUS1	Pseudouridine synthase 1
Q	Queuosine
QTRT1	Queuine trna-ribosyltransferase catalytic subunit 1
QTRT2	Queuine trna-ribosyltransferase auxiliary subunit 2
RNA	Ribonucleic acid
ROS	Reactive oxygen species
rRNA	Ribosomal RNA
s^2^U	2-thiouridine
SAA1	Serum amyloid A1
scRNA-seq	Single-cell RNA sequencing
SELENON	Selenoprotein N
SLC7A7	Solute carrier family 7 member 7
SMAD2	SMAD family member 2
SMO	Smoothened
snoRNA	Small nucleolar RNA
snRNA	Small nuclear RNA
snRNA-seq	Single-nucleus RNA sequencing
SOD1	Superoxide dismutase 1
SP2	Transcription factor Sp2
STK11	Serine/threonine kinase 11
t^6^A	N^6^-threonylcarbamoyladenosine
TDP-43	TAR DNA-binding protein 43
TET	Ten-eleven translocation
TET1	Tet methylcytosine dioxygenase 1
TETs	Ten-eleven translocation proteins
TGFβ	Transforming growth factor-beta
TGFβ1	Transforming growth factor beta 1
TGS1	Trimethylguanosine synthase 1
TLR4	Toll-like receptor 4
TM4SF1	Transmembrane 4 L six family member 1
TMG	Trimethylguanosine
TNNT	Troponin T
TRMT1	Trna methyltransferase 1
TRMT10A	Trna methyltransferase 10A
TRMT11	Trna methyltransferase 11
TRMT2A	Trna methyltransferase 2A
TRMT5	Trna methyltransferase 5
TRMT6	Trna methyltransferase 6
TRMT61A	Trna methyltransferase 61A
TRMU	Trna 5-methylaminomethyl-2-thiouridylate methyltransferase
tRNA	Transfer RNA
TYW1	Trna-yw synthesizing protein 1
TYW2	Trna-yw synthesizing protein 2
TYW3	Trna-yw synthesizing protein 3
TYW4	Trna-yw synthesizing protein 4
UPS	Ubiquitin-proteasome system
USP1	Ubiquitin specific peptidase 1
UTR	Untranslated region
WDR4	WD repeat domain 4
WTAP	WT1 associated protein
YBX1	Y-box binding protein 1
YBX2	Y-box binding protein 2
YTHDC1	YTH domain containing 1
YTHDF1	YTH N^6^-methyladenosine RNA binding protein 1
YTHDF2	YTH N^6^-methyladenosine RNA binding protein 2
YTHDF3	YTH N^6^-methyladenosine RNA binding protein 3
yW	Wybutosine
τm^5^s^2^U	5-taurinomethyl-2-thiouridine
