# Correction: Chen et al. RNA Chemical Modifications in Mammalian Skeletal Muscle Development, Homeostasis, and Disease: Regulatory Mechanisms and Chemical Biology Perspectives. *Molecules* 2026, *31*, 2797

**DOI:** 10.3390/molecules31183294

**Published:** 2026-09-17

**Authors:** Dujun Chen, Mailin Gan, Yuhang Lei, Xinyi Wang, Jincheng Zan, Qing Ye, Xiaofeng Zhou, Lei Chen, Yan Wang, Ye Zhao, Li Zhu, Linyuan Shen

**Affiliations:** 1 Farm Animal Germplasm Resources and Biotech Breeding Key Laboratory of Sichuan Province, Sichuan Agricultural University, Chengdu 611130, China; 2024202034@stu.sicau.edu.cn (D.C.); ganmailin@sicau.edu.cn (M.G.); leiyuhng@gmail.com (Y.L.); 2024202054@stu.sicau.edu.cn (X.W.); zanjincheng@stu.sicau.edu.cn (J.Z.); yeqing@stu.sicau.edu.cn (Q.Y.); zxf93715@163.com (X.Z.); chenlei815918@sicau.edu.cn (L.C.); 14916@sicau.edu.cn (Y.W.); zhye@sicau.edu.cn (Y.Z.); 2 State Key Laboratory of Swine and Poultry Breeding Industry, Sichuan Agricultural University, Chengdu 611130, China; 3 Key Laboratory of Livestock and Poultry Multi-Omics, Ministry of Agriculture and Rural Affairs, College of Animal and Technology, Sichuan Agricultural University, Chengdu 611130, China

## Text Correction

There was an error in the original publication [1]. The last sentence of the third paragraph in Section 2.1. “ADAR proteins mediate the fate transformation of muscle-derived cells by regulating the structural stability of specific lncRNAs [30]” is incorrect and has been removed. 

The following sentence is correct and has been added to the end of the first paragraph in Section 2.1.

**The HNRNPA2B1 protein mediates the fate transition of myogenic cells by regulating specific intrinsic and downstream splicing regulators [30].**

The authors state that the scientific conclusions are unaffected. This correction was approved by the Academic Editor. The original publication has also been updated.

## References

[1] Chen D., Gan M., Lei Y., Wang X., Zan J., Ye Q., Zhou X., Chen L., Wang Y., Zhao Y. (2026). RNA Chemical Modifications in Mammalian Skeletal Muscle Development, Homeostasis, and Disease: Regulatory Mechanisms and Chemical Biology Perspectives. Molecules.

